# Distinct seasonality and increased respiratory failure in RSV patients < 2 years of age after emergence of SARS-CoV-2: data from the multicentric, prospective PAPI study

**DOI:** 10.1007/s00431-025-06057-0

**Published:** 2025-03-13

**Authors:** Jessica Bähre, Matthias Lange, Patrick Salaschek, David Twardella, Stefan Arens, Frank Eberhard, Grit Barten-Neiner, Marcus Panning, Holger Köster, Cordula Körner-Rettberg, Martin Wetzke, Christine Happle

**Affiliations:** 1 Children’s Hospital Auf Der Bult, Hannover, Germany; 2 Department of Pediatrics, Elisabeth Children’s Hospital, University of Oldenburg, Oldenburg, Germany; 3 Pediatric Pneumology, Children’s Hospital at Marien-Hospital Wesel, Wesel, Germany; 4 Pediatric Department, Children’s Hospital at Marien-Hospital Vechta, Vechta, Germany; 5 CAPNETZ Stiftung, Hannover, Germany; 6 Faculty of Medicine, Institute of Virology, Medical Center, University of Freiburg, Freiburg, Germany; 7 Department of Pediatric Pneumology, Allergy, and Neonatology, Hannover Medical School, Hannover, Germany; 8 German Center for Lung Research, Biomedical Research in End-Stage and Obstructive Lung Disease Hannover (BREATH), Hannover, Germany; 9 Excellence Cluster on Infection Research “Resolving Infection Susceptibilily” RESIST, Hannover, Germany

**Keywords:** RSV, Resurgence, COVID-19, Pandemic, Seasonality

## Abstract

Respiratory syncytial virus (RSV) is a leading cause for global infant morbidity and mortality. The COVID-19 pandemic caused significant shifts in seasonality of RSV, and changes in disease severity have been matter of intense discussion. Between September 2020 and February 2023, the multicentric, prospective PAPI study analyzed rates and phenotypes of hospitalized RSV patients aged ≤ 24 months across three German hospitals. Pseudonymized patient data were analyzed employing Mann–Whitney *U* and chi-square testing, or one-way ANOVA or Kruskal–Wallis testing when more than two groups were compared. Additionally, RSV cases from seasons 2017/2018–2020/2021 were retrospectively analyzed. After its absence in 2020/2021, RSV returned approximately 2 months earlier than usual in late 2021. Overall duration of the season and patient numbers were comparable to previous seasons, and no significant shifts in age and gender distributions occurred in our cohort. While duration of hospitalization did not differ between the periods before vs. after the emergence of SARS-CoV-2, a significantly higher rate of patients with hypoxemia and respiratory failure occurred after the onset of the pandemic (oxygen supplementation post vs. pre: 59.4% vs. 54.8%, *p* < 0.001, non-invasive ventilation post vs. pre: 12.4% vs. 7.2%; *p* < 0.001). No deaths occurred during the entire observational period.

*Conclusion*: We present comprehensive data on distinct seasonality and increased disease severity in children hospitalized with RSV bronchiolitis before and after the onset of the SARS-CoV-2 pandemic. Our data aids in understanding the impact of the pandemic on RSV disease in infants and provides valuable information on the impact of RSV on pediatric healthcare prior to broad introduction of novel prevention measures such as nirsevimab.

**Table:** 

**What is Known:** • *Respiratory syncytial virus (RSV) is a leading cause of infant morbidity and mortality globally*. • *COVID-19 has led to significant shifts in RSV seasonality, and concerns about shifts in RSV severity*.
**What is New:** • *This study shows distinct seasonality and significant shifts in diseases severity amongst children with RSV associated hospitalization under the age of 2 yrs in the last years in Germany*. • *It reports significantly higher rates of RSV associated respiratory failures in children < 2 yrs. of age after emergence of the pandemic*.

**Supplementary Information:**

The online version contains supplementary material available at 10.1007/s00431-025-06057-0.

## Introduction

Globally, respiratory syncytial virus (RSV) belongs to the most frequently identified pathogen in infants and toddlers with acute lower respiratory infections (LRTI) [1, 2]. RSV contributes substantially to worldwide childhood morbidity and mortality: an estimated 1/50 of deaths in children aged 0–60 months and 1/28 deaths in children aged 1–6 months are attributable to RSV [3]. Although RSV mortality rates are higher in low-income countries, also in middle- to high-income countries, RSV prevalence is associated with significant healthcare burden [3, 4]. RSV follows a distinct seasonal pattern [5]. In the northern hemisphere, e.g., in Europe and the United States of America (USA), RSV bronchiolitis typically occurs between November and March [6]. However, non-pharmaceutical interventions (NPI) such as face masks and social distancing that have been implemented to control the SARS-CoV-2 pandemic induced significant shifts in both frequency and seasonality of other non-SARS-CoV-2 airway pathogens [7–9]. RSV was virtually absent during winter 2020/2021, and many authors hypothesized a lack of natural immunity to RSV in infants during the pandemic, associated with altered seasonality, increased disease severity, and stress on healthcare systems upon lifting of the measures to restrict SARS-CoV-2 transmission [10–12]. Another factor strongly impacting frequency and severity of airway infections in children is vaccinations. For RSV, new and effective prevention strategies have been recently introduced [13–15].

To study the impact of such factors on RSV frequency and severity in young children and to comprehensively describe the burden of virus-associated LRTI in infants and toddlers in Germany, we established the Pediatric Airway Pathogen Incident (PAPI) study. Our current analyses aimed at comparing RSV seasonality and clinical phenotypes in children until the age of 2 years in the phases before vs. after the emergence of COVID-19, hypothesizing increased RSV disease severity after emergence of COVID-19. Having noted the absence of RSV bronchiolitis during the fall/winter season 2020/2021 in Germany [16], we here show that RSV recurred with earlier seasonal appearance in the following years. We deliver comprehensive data on RSV disease severity comparing seasons before and after the emergence of SARS-CoV-2 and present detailed information on phenotypes and risk profiles of affected infants and toddlers throughout RSV seasons since the fall of 2017.

## Methods

### Prospective data analysis

PAPI is a multicentric, prospective study analyzing the incidence of viral pathogens and clinical phenotypes of infants and toddlers ≤ 24 months of age hospitalized with lower respiratory tract infections (LRTI) in Germany [16]. Prospective data analysis for the here-described cohort started in September 2020 and ended in February 2023. During this period, palivizumab vaccinations in Germany were regularly applied to children at high risk of severe disease courses upon early RSV infection during their first RSV seasons [17]. Of note, the market entry of nirsevimab in Germany was in fall 2023, hence succeeded the analyzed time period [18]. The measures to prevent the spread of COVID-19 (e.g., social distancing, daycare center closure) had been significantly lifted throughout 2020, e.g., marked by the gradual reopening of childcare centers to the public in the spring of 2022 [19]. Three pediatric centers contributed cases: Hannover Medical School, Elisabeth Children’s Hospital University of Oldenburg, and Marien-Hospital Wesel. In each center, a systematic screening of all infants ≤ 24 months old was conducted to identify those displaying the following signs and symptoms (case definition): *Symptom group A*, fever, cough, rhinitis, and pharyngitis, and *Symptom group B*, wheezing, crackles, attenuated breath sounds, tachypnea, dyspnea, and hypoxemia. For study inclusion, at least one clinical feature from each symptom group had to be met. Once included, all probands underwent nasopharyngeal swabbing with consecutive polymerase chain reaction (PCR) testing for RSV and other respiratory viruses.

### Retrospective data analysis

Complementing prospective data analyses, a retrospective analysis of all hospitalized RSV bronchiolitis patients since the start of the typical RSV season in 2017/2018 (in pre-pandemic years usually starting between calendar week 44–50 [20]) was conducted in the three participating centers. In all centers, systematic RSV screening via rapid antigen or PCR testing for all infants hospitalized with signs of LRTI had been in place as a standardized operational procedure already prior to 2017. Additionally, these centers had installed systematic digital data collection of clinical parameters and RSV testing since before 2017. For retrospective data analyses, the same case definition was applied as for the prospective analysis, and data was extracted from hospital databases.

### Statistical analysis

Before inclusion into a central database, all data was pseudonymized. It was then analyzed using Microsoft Excel (version 2019), SPSS Statistics (version 20), and GraphPad Prism (version 5). We assessed the shift in peak seasonality visually by identifying the week with the most RSV cases per recorded season. Rates (e.g., percentage of children receiving ventilatory support) were calculated by assessing the proportion of children with documented application of such treatment (e.g., non-invasive or invasive ventilation (NIV or IV)) within the cohort. To assess statistical differences between the two groups, categorical items were compared using chi-square testing, and differences in numerical items were assessed using *T*-test (normally distributed data) or Mann–Whitney *U* testing (for not normally distributed data). When more than two groups were compared, one-way ANOVA (for data following normal/Gaussian distribution) or Kruskal–Wallis with post hoc Dunn’s testing (for data not normally distributed) were applied. The significance level *α* was set to a *p* value of 0.05.

### Ethics

All study procedures were conducted in accordance with the Declaration of Helsinki, and all institutional review committees (local medical ethics committees) of participating centers approved the study protocol (ethical approval Hannover Medical School #9442_BO_K_2020 MHH). For prospective data collection and analyses, legal guardians of all participants gave their written informed consent.

## Results

We collected data from all hospitalized children ≤ 24 months with LRTI from RSV seasons 2017/2018 to 2022/2023. In total, data from *n* = 898 RSV cases (*n* = 330 prospective, *n* = 568 retrospective cases) were included in the analysis.

**Fig. 1:**
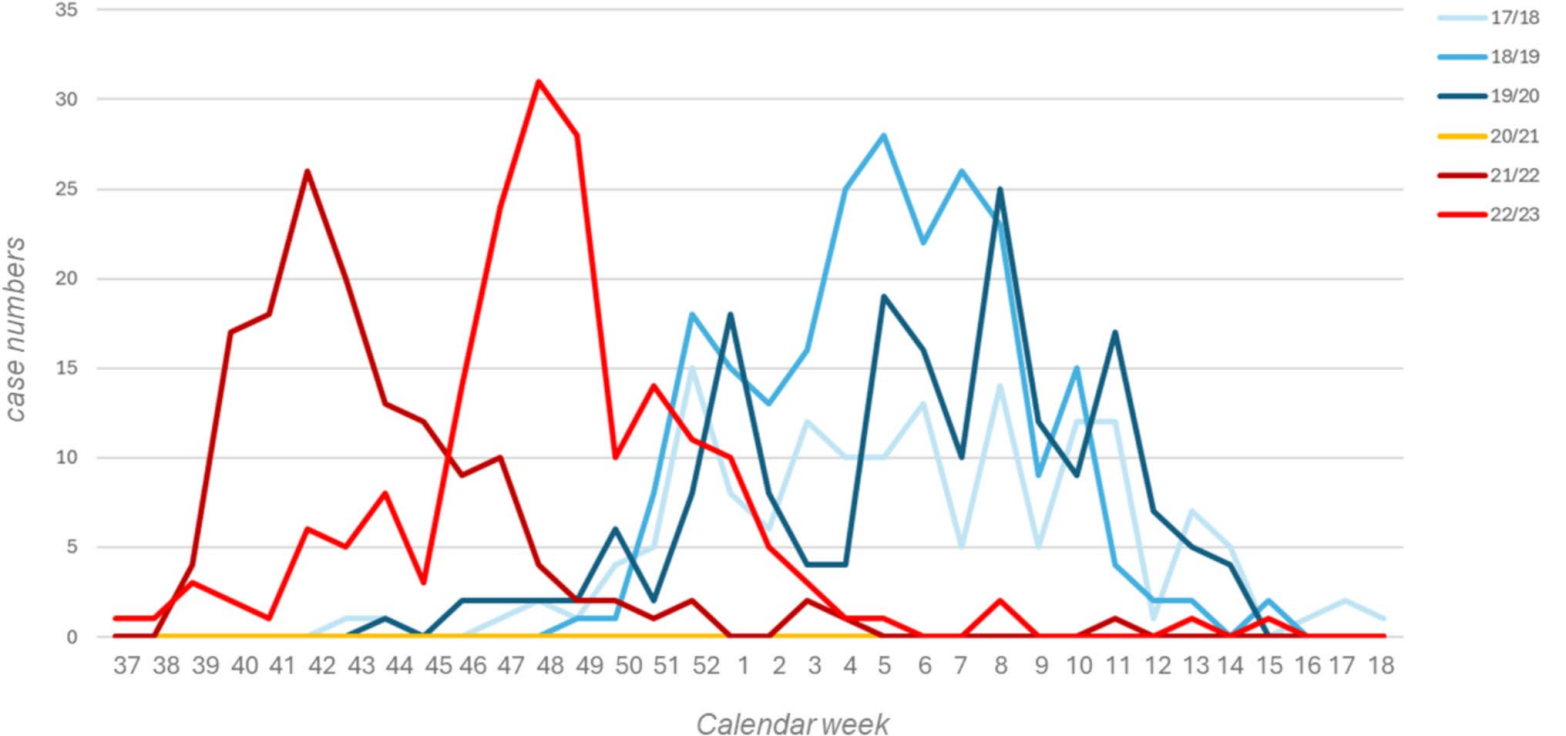
Number of patients per calendar week across seasons

As shown in Fig. 1, the absence of RSV in the first fall/winter season after emergence of SARS-CoV-2 (yellow flatline) was followed by an early, atypical RSV season in 2021/2022 (dark red line). In 2021, the first RSV cases occurred during calendar week 38, around 12 weeks before the typical pre-pandemic start of RSV. The peak of RSV-associated hospitalizations after the first resurge of this virus during the pandemic occurred in late 2021, around calendar week 41. This was approximately 3.5 months earlier than previous typical RSV hospitalization peaks. This phenomenon was observed independently in all centers and was in line with a national survey on RSV hospitalizations (Suppl. Figure 1). In the following year (season 2022/2023, orange line, Fig. 1), the peak of RSV-related hospitalizations had shifted, around 7 weeks into the direction of pre-pandemic surges. Overall, the durations of RSV seasons 2021/2022 and 2022/2023 were comparable to pre-pandemic seasons. Also, patient numbers per season did not differ significantly in pre- vs. post-emergence of SARS-CoV-2 seasons (median 65 cases vs. 61 cases per center and season, *p* 0.9).

**Fig. 2:**
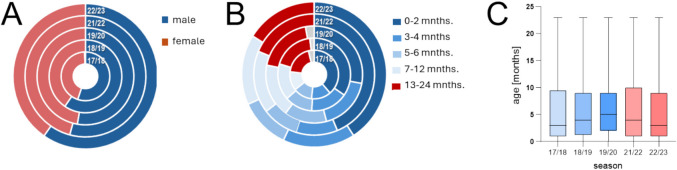
Sex (**A**) and age group (**B**) distribution across seasons. Median ages of patients in the analyzed seasons (**C**). (Donut pies in **A** and **B** show the distribution of items per season in concentrical circles; bars in **C** display median with box and whiskers)

Next, we analyzed the age, sex, and risk profiles of RSV patients hospitalized before versus after emergence of SARS-COV-2. Across all seasons, more boys than girls were noted (Fig. 2A), and no significant changes in sex distribution occurred (56.2% males pre- vs. 57.3% post-onset of the pandemic, *p* 0.8). Similarly, age profiles were comparable across all seasons (Fig. 2B). Throughout all seasons, the majority of patients were infants below the age of 1 year (pre- vs. post-emergence of SARS-CoV-2: 78.5% vs. 80.9% *p* 0.4). Also, regarding the distribution of other age groups and ages across seasons (Fig. 2C) and in pre-pandemic vs. later seasons, no significant changes were observed (median age pre vs. post: 4 vs. 3.5 months, *p* 0.1).

**Fig. 3:**
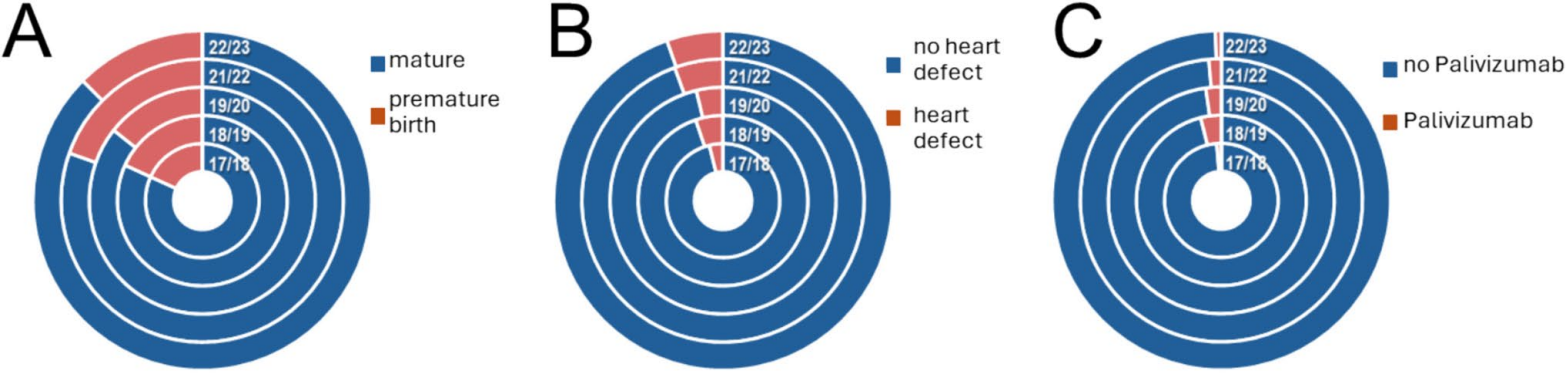
RSV risk factors and palivizumab treatment rates across seasons. **A** Prematurity (< 37.0 weeks of gestation). **B** Rates of heart defect and **C** palivizumab treatment rates across seasons (donut pies display distribution of items per season in concentrical circles)

Across all seasons, around one quarter of children were prematurely born (Fig. 3A), and no significant differences with regard to prematurity rates among RSV-associated hospitalizations were observed in pre- vs. post-onset of the pandemic (17.1% vs. 13.6%, *p* 0.2). The frequency of children with cardiac defects was highest in the first season after emergence of SARS-CoV-2 (5.6% in 2021/2022) and lowest in the first season of our observational period (2017/2018), and a slightly higher rate of heart defects was observed when we compared all patients from pre- vs. all of those recruited post-emergence of SARS-CoV-2 (Fig. 3B, 4.4% vs. 5.5% of hospitalizations before vs. after onset of the pandemic, *p* 0.5). The rate of patients that had received palivizumab was lowest in the last season, but overall vaccination rates in pre- vs. post-start of the pandemic were not significantly different (2.6% vs. 0.9%, *p* 0.1). During the observational period, no patient had received novel RSV vaccinations such as nirsevimab, and no mother had received RSV vaccinations during pregnancy.

To assess potential changes in RSV disease severity after the advent of SARS-CoV-2, we additionally analyzed healthcare provision data within the cohort.

**Fig. 4:**
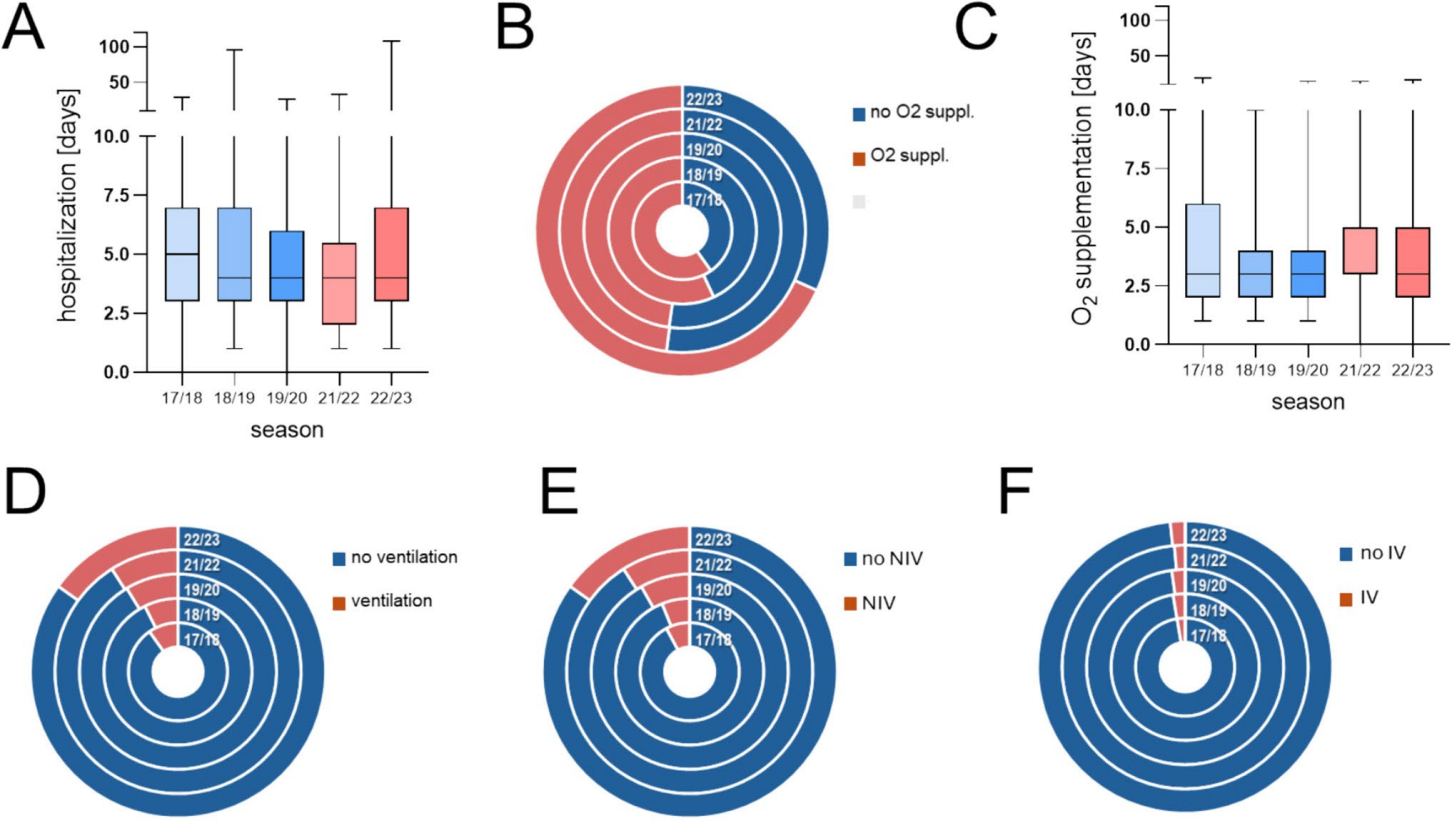
Disease severity and treatment demand across seasons. **A** Duration of hospitalization. **B** Rates of and **C** duration of oxygen supplementation. **D** Rates of ventilation and **E**, **F** rates of non-invasive (NIV) and invasive (IV) ventilation (bars in **A** and **C** display box and whiskers; donut pies in **B** and **D**–**F** display distribution of items per season in concentrical circles)

As shown in Fig. 4A, hospitalization lengths were not significantly different when comparing each season separately. Also when comparing all patients recruited after the emergence of SARS-CoV-2 in comparison to all those from pre-pandemic seasons, the mean duration of hospitalization was not significantly different between these periods (pre vs. post: 5.4 vs. 5.2 days, *p* 0.6). The rate of oxygen supplementation was highest in the last season (2022/2023, Fig. 4B), and significantly more children received supplemental oxygen in the period after the onset of the pandemic than in the time before (59.4% (post) vs. 54.8% (pre), *p* < 0.001). When comparing oxygen supplementation courses between individual seasons, durations did not differ significantly between them (Fig. 4C), and also, the comparison of oxygen supplementation durations before vs. after advent of SARS-CoV-2 was comparable (median 3 vs. 3 days, *p* 0.5).

During the analyzed period, the rate of patients requiring ventilation ranged from 6.1% in 2018/2019 to 15.1% in 2022/2023 (Fig. 4D), with significantly higher rates of ventilation in the last seasons (pre- vs. post-onset of the pandemic: 8.5% vs. 12.7%, *p* 0.05). This difference was mainly driven by the significantly higher rates of NIV (continuous positive airway pressure or high flow ventilation) in the time after emergence of SARS-CoV-2 (NIV in post vs. pre: 12.4% vs. 7.2%; *p* < 0.001). Invasive ventilation was rare in both periods, with no significant difference between pre- vs. post-emergence of COVID-19 (2.5% vs. 1.5%; *p* 0.5). Among the subset of RSV patients receiving NIV or IV, no significant changes in ventilation durations occurred between individual seasons (Suppl. Figure 2). We also found no significant difference when comparing all patients from pre- vs. all cases from post-onset of the pandemic (median days pre- vs. post-emergence of COVID-19; NIV: 4 vs.4 days, *p* 0.8; IV: 8 vs. 5.5, *p* 0.5). No deaths occurred during the entire period of analysis.

## Discussion

The COVID-19 pandemic has led to significant shifts in the seasonality of infectious pathogens [8, 9, 21–24]. Following the repeal of non-pharmaceutical interventions to restrict the spread of COVID-19—measures such as masking, social distancing, and daycare and school closures—an increase in RSV-related hospitalization rates among preschool children was observed [25, 26]. On this basis, it was hypothesized that case numbers and disease severity during “comeback seasons” of RSV might surpass those in previous RSV seasons [5, 27], assumingly due to reduced immunity against RSV during the pandemic [28]. Our data aids in understanding the impact of the pandemic on pediatric airway pathogens as it compares RSV seasonality and clinical phenotypes in severely affected infants throughout the phases before, during, and after the emergence of COVID-19.

We present comprehensive data from three German hospitals with structured data collection across five seasons from before vs. after the emergence of SARS-CoV-2. After reporting the absence of the typical RSV season during fall/winter 2020/2021 in Germany [16], we now demonstrate a significant shift in RSV seasonality and increase in disease severity after lifting of the non-pharmaceutical interventions in subsequent seasons.

In the two seasons after RSV resurgence, durations and numbers of RSV-associated hospitalization were comparable to previous seasons, but significantly higher rates of hypoxemia and respiratory failure (need for ventilatory support) occurred. We observed significantly more children with demand for oxygen supplementation, and more children received ventilatory support through high flow oxygen therapy and continuous positive airway pressure ventilation. Our finding that increased treatment demand (more oxygen supplementation and ventilatory support) in the last seasons was not associated with increased hospitalization times was somewhat surprising. Possibly, limited hospitalization capacities in the pediatric centers during the resurgence of RSV warranted more stringent discharge procedures for children after termination of oxygen supplementation. However, our analysis is not suited to analyze this question, and we can only speculate on the reasons for this observation. Taken together, our data suggests more severe RSV phenotypes after resurgence of RSV in 2021. As such, our data support the hypothesis of other authors that RSV returned with more severe disease severity in infants and toddlers upon lifting of isolation measures, possibly due to post-pandemic “RSV immunity debt” [7, 8, 11, 21]. While our data illustrates increased treatment demand in the era after reemergence of RSV, it is not suited to address the question of “immunity debt.” Interestingly, however, recent analyses show no significant shifts in RSV-neutralizing antibody levels following the measures to contain COVID-19, and as such do not support the notion that a humoral “immunity debt” led to increased morbidity in RSV seasons following the pandemic [29, 30].

We did not observe a significant increase in case numbers per season since 2021. This is in contrast to recently published analyses from Germany and Denmark showing higher rates of RSV-related hospitalizations in small children in 2021/2022 as compared to pre-pandemic times [31, 32]. The most relevant reason for this discrepancy may lie in the design of our study. Firstly, we recruited only children up to the age of 2 years, undoubtedly the age group with the largest burden for severe RSV disease courses [33]. Another study reported an increased case number of severe RSV post-emergence of COVID-19; however, it also included children up to the age of 5 years and observed the highest increase in 3- and 4-year-old children [32], not in newborns and infants that our study focused on. Secondly, the highly stringent screening for RSV that had been installed in the PAPI study centers already before the onset of the pandemic has led to an accurate registration of all cases already prior to the start of the pandemic. Some authors suggested that higher registrations of RSV cases after the emergence of COVID-19 may be attributed to increased rates of RSV testing [34], which could also have impacted recorded case numbers in Denmark and beyond.

Our findings on the altered RSV seasonality are in concordance with a plethora of other reports documenting the early and out-of-season recurrence of RSV in Europe and elsewhere in 2021/2022 [7, 8, 31, 34–36]. Notably, the pattern of the RSV season 2021/2022 observed in our study is perfectly in line with data from a national ad hoc survey on RSV hospitalizations in Germany, which was implemented approximately 1 year after our prospective data collection began [26].

With regard to the rate of classic RSV risk factors, we observed no significant shifts after the emergence of SARS-CoV-2, but a lower proportion of patients that had received palivizumab in the two seasons after the emergence of COVID-19. While this difference was not significant and we cannot assess causality in this finding, this could support the notion of insufficient RSV vaccination coverage in seasons 2021/2022 and 2022/2023. In Germany, vaccination recommendations were adjusted to the premature RSV resurgence in late 2021, and it was advised to begin palivizumab immunizations in calendar week 39 [37]. In our observation, hospitalizations due to RSV began to surge around calendar week 38, and given the natural lack between official recommendation and its actual clinical implication, one can assume that vaccinations to premature infants had not yet been sufficiently implemented by the time the RSV season had fully begun.

Our observation of the high RSV-associated treatment burden is in line with a plethora of previous publications and relevant in the context of the newly approved RSV vaccines [13, 15, 38]. Our data illustrate the high RSV-associated morbidity of infants in Germany and illustrate the need for effective prevention. Data from Spain and the USA demonstrated that the burden of severe RSV with the need for hospital care can be reduced by 70 to 80% after appropriate RSV vaccination coverage [14, 39]. Based on its comprehensive, long-standing, and real-world approach, PAPI can and will assess the impact of novel RSV immunization programs on the frequency and severity of RSV-associated hospitalizations in infants and toddlers in Germany.

Our study has several important limitations. Firstly, our prospective data collection started in the fall of 2020, and although our analyses of previous RSV seasons were conducted exclusively in centers with systematic RSV surveillance and thorough and systematic clinical data collection, the data we obtained before fall 2020 may be incomplete due to its retrospective nature. Furthermore, although we deliver multicentric, prospective clinical data on RSV bronchiolitis from three large pediatric hospitals in Germany, these centers represent only a fraction of hospitals involved in the care of children with RSV. We started case registrations in the late summer of each year but may have missed atypical RSV cases occurring earlier in each year. Also, the PAPI study only included hospitalized children until the age of 2 years of age. RSV, however, is also associated with significant morbidity in ambulatory care settings, and in older children as well as adults and seniors [40–43]. In spite of these limitations, we believe our data is representative as it strongly concurs with that from the RSV surveillance network established in late 2021 by the German Society for Pediatric Infectious Diseases [26]. Our study focused on severely ill RSV patients ≤ 24 months of age and did not include information on older children. However, recent data from Germany suggests that under 20% of RSV infections leading to hospital admission affect children above the age of 2 years [26].

Despite its limitations, our work provides important information on RSV-associated morbidity in Germany. Our study presents comprehensive, multicentric, and prospectively collected data on phenotypes and risk profiles of children hospitalized with RSV bronchiolitis before and after the emergence of SARS-CoV-2. We demonstrate distinct seasonality and increased disease severity compared to the pre-pandemic period. We also provide valuable information on the impact of RSV on pediatric healthcare prior to the broad implementation of novel prevention measures such as nirsevimab. As such, we hope our work helps in preparing for future, atypical waves of airway pathogens and to evaluate and adapt prevention strategies against RSV in Germany and beyond.

## Supplementary Information

Below is the link to the electronic supplementary material.

Supplementary file1 (DOCX 175 KB)

## Data Availability

Availability of data and material The data and biomaterials underlying our analysis may be requested by submitting a formal request to the study board, who will evaluate it. Email requests may be directed to wetzke.martin@mh-hannover.
