# Supplementary material for: Distinct seasonality and increased respiratory failure in RSV patients < 2 years of age after emergence of SARS-CoV-2: data from the multicentric, prospective PAPI study
Source: Eur J Pediatr. 2025 Mar 13;184(4):246. doi: 10.1007/s00431-025-06057-0 (PMC11906531; doi:10.1007/s00431-025-06057-0)
Supplement: Supplementary file 1 — Supplementary file1 (DOCX 175 KB) [file 431_2025_6057_MOESM1_ESM.docx]

**Supplementary information
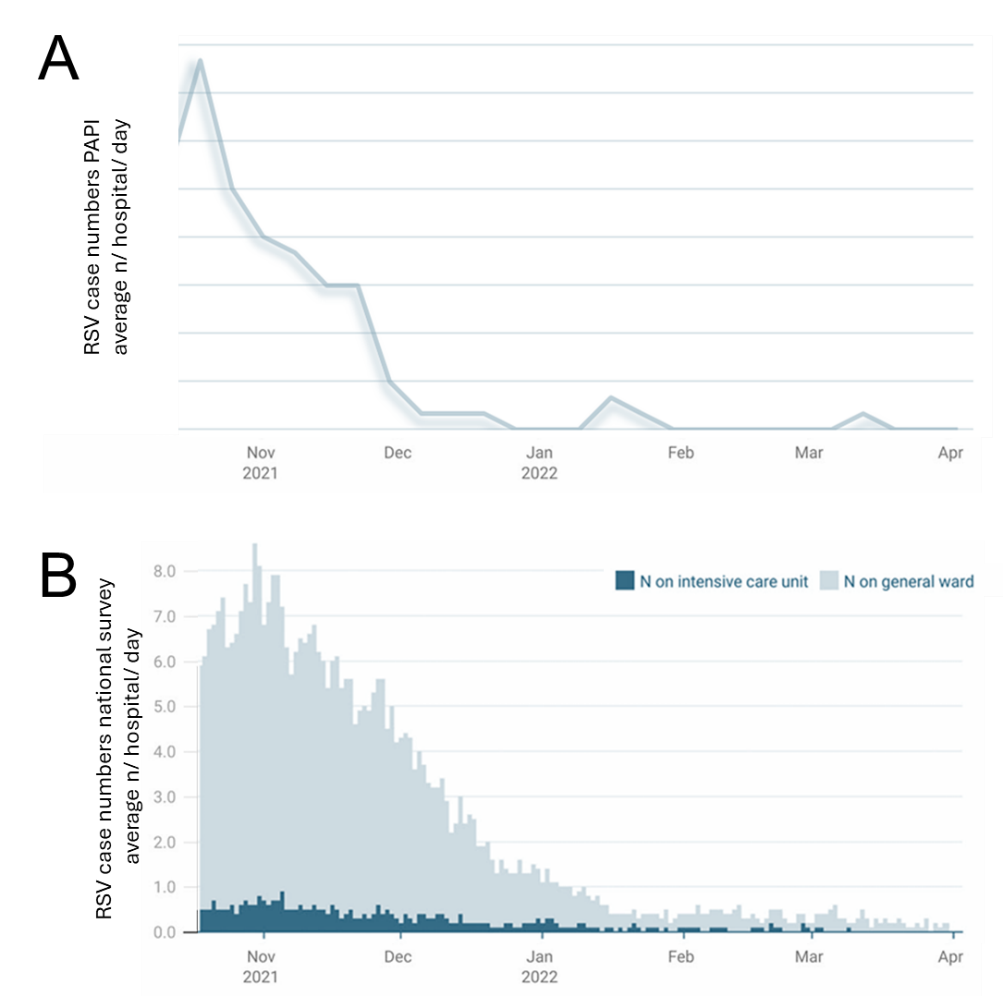
**

*Suppl. Fig. 1: Comparison of RSV seasonality in participating PAPI centers and across Germany. [A] RSV seasonality in our study (season 2021/2022, average cases per hospital) as compared to [B] national ad hoc survey data on hospitalized RSV cases (Figure adapted from [1]).*

**´**

**
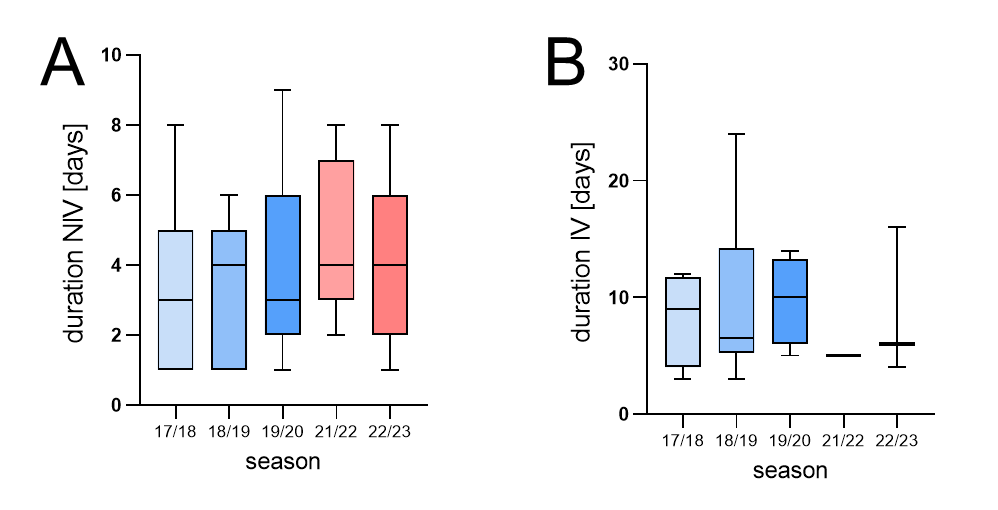
**

*Suppl. Fig. 2: [A] Duration of hospitalization non-invasive (NIV) and [B] rates of invasive (IV) ventilation [bars display box and whiskers].*

**Supplementary Reference**

1. Tenenbaum T, Doenhardt M, Diffloth N, Berner R, Armann JP: **High burden of RSV hospitalizations in Germany 2021-2022**. *Infection* 2022, **50**(6):1587-1590.
